# Language-Selective and Domain-General Regions Lie Side by Side within Broca’s Area

**DOI:** 10.1016/j.cub.2012.09.011

**Published:** 2012-11-06

**Authors:** Evelina Fedorenko, John Duncan, Nancy Kanwisher

**Affiliations:** 1 McGovern Institute for Brain Research and Brain and Cognitive Sciences Department, Massachusetts Institute of Technology, 43 Vassar Street, Cambridge, MA 02139, USA; 2 Cognition and Brain Sciences Unit, Medical Research Council, 15 Chaucer Road, Cambridge CB2 7EF, UK

## Abstract

In 1861, Paul Broca stood up before the Anthropological Society of Paris and announced that the left frontal lobe was the seat of speech. Ever since, Broca’s eponymous brain region has served as a primary battleground for one of the central debates in the science of the mind and brain: Is human cognition produced by highly specialized brain regions, each conducting a specific mental process, or instead by more general-purpose brain mechanisms, each broadly engaged in a wide range of cognitive tasks? For Broca’s area, the debate focuses on specialization for language versus domain-general functions such as hierarchical structure building (e.g., [1, 2]), aspects of action processing (e.g., [3]), working memory (e.g., [4]), or cognitive control (e.g., [5–7]). Here, using single-subject fMRI, we find that both ideas are right: Broca’s area contains two sets of subregions lying side by side, one quite specifically engaged in language processing, surrounded by another that is broadly engaged across a wide variety of tasks and content domains.

## Highlights

► Broca’s area contains both language-selective and domain-general subregions ► “Broca’s aphasia” plausibly results from damage to both sets of subregions ► These data help reconcile competing views of Broca’s area ► Human cognition is produced by both specialized and general-purpose brain regions

## Results and Discussion

Despite abundant theorizing and extensive empirical investigation, active debates continue between contrasting language-specific (e.g., [8–10]) and domain-general (e.g., [1–7]) views of Broca’s area. To test these alternatives, we conducted an fMRI experiment in which we first scouted within traditionally defined Broca’s area in individual subjects for candidate language-selective voxels and candidate domain-general voxels. We then tested each set of voxels more stringently across six subsequent experiments designed to quantify their engagement in a set of nonlinguistic functions that have been previously attributed to Broca’s area.

Subjects read sentences and lists of nonwords, each followed by a probe test of recognition memory (present/absent judgment on a single probe word/nonword). To search for language-responsive regions within Broca’s area, we asked whether any voxels within left-hemisphere Brodmann areas (BAs) 44 and 45 [11] responded significantly more strongly to sentences than to nonword lists [12]. To search for regions sensitive to broad cognitive demands [7], we used the opposite contrast (nonwords > sentences) because the nonword task is substantially and significantly more difficult than the sentence task [12]. To further characterize any brain regions fitting either profile, in separate runs participants performed tasks tapping mental arithmetic, spatial and verbal working memory, and several varieties of cognitive control. Each task had a harder and an easier condition, and all had been previously reported to elicit activity in or near Broca’s area.

**Figure 1:**
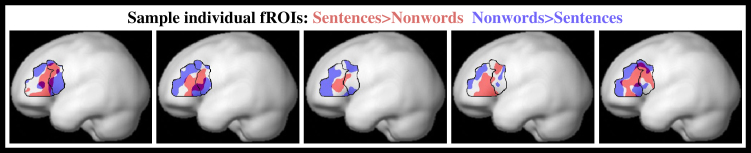
Activation Patterns for Five Example Subjects Individual subject activations (red: sentences > nonwords; blue: nonwords > sentences; threshold: p < 0.001, uncorrected; the apparent overlap at the edges of the regions of interest [ROIs] results from the 3D projection of independent regions that overlap along the line of sight). These activations served as the ROIs. Black outlines show BA45/44 borders [11].

**Figure 2:**
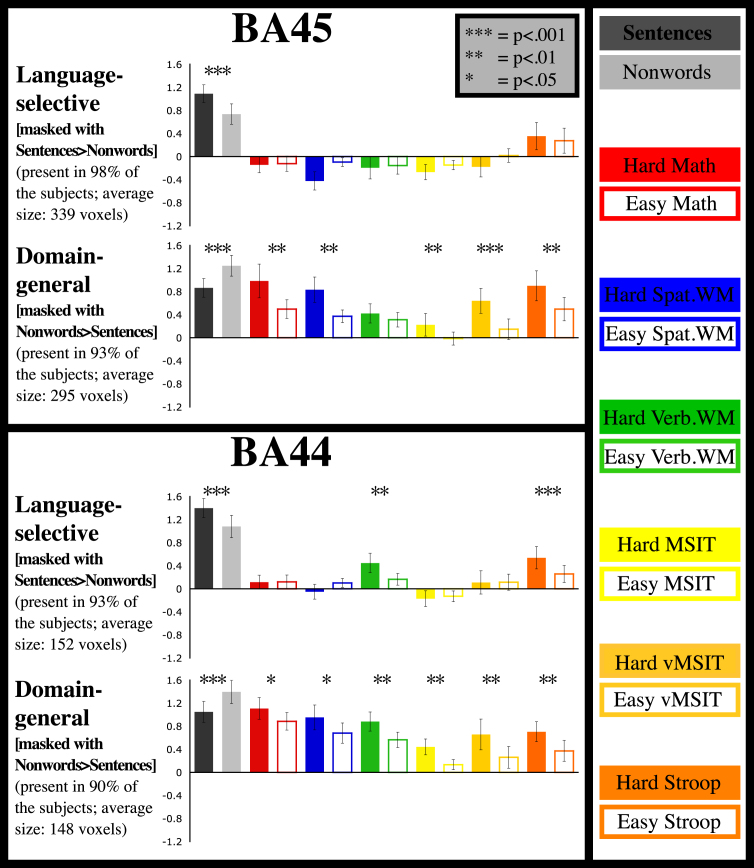
Functional Profiles of Language-Selective and Domain-General Functional ROIs Magnitude of response (in percent signal change from the fixation baseline) of language-selective and domain-general regions within BA45 (top box) and BA44 (bottom box) to each of the two conditions in each of the seven tasks. Language-selective regions are defined by intersecting BA45/44 with sentences > nonwords activation, and domain-general regions are defined by intersecting BA45/44 with nonwords > sentences activation. All magnitudes shown are estimated from data independent of those used to define the regions; responses to the sentences and nonwords are estimated using a left-out run. Error bars represent SEM by participants. ^∗^p < 0.05; ^∗∗^p < 0.01; ^∗∗∗^p < 0.001. In the math task, participants added smaller versus larger numbers; in the spatial and verbal working memory (WM) tasks, participants kept in memory fewer versus more locations or digits, respectively; and in the three cognitive control tasks (MSIT, vMSIT, Stroop), participants had to inhibit a prepotent but task-irrelevant response and choose instead the task-relevant response.

We found two distinct subregions, each present in at least 90% of subjects individually, with strikingly different but highly reliable functional profiles, lying side by side within Broca’s area and each spanning the BA44/45 boundary (Figure 1). One subregion, identified in each subject as responding more during the processing of sentences than nonwords, showed little response to any other task, with the BA45 portion being particularly selective (Figure 2, top row; see also Table 1). As we have reported previously based on partially overlapping data [13], this region is part of a broad left-hemisphere language-selective network, with additional major components in left temporal and parietal lobes. In contrast, another set of subregions exhibited extreme domain generality (Figure 2, bottom row; see also Table 1), with a greater response to the harder than the easier condition in each of the seven tasks, regardless of stimulus (verbal/nonverbal) and task (arithmetic/working memory/inhibition). Our data thus provide seven statistically independent replications of this same hard > easy contrast across diverse cognitive domains.

Despite some variability across individuals (Figure 1), the topography of these subregions is remarkably consistent: domain-general subregions abut the language-selective subregion posteriorly (extending toward the inferior precentral sulcus), dorsally (extending to the inferior frontal sulcus), and ventrally. Within traditionally defined Broca’s area (Figure 1, black outlines), accordingly, our results show a distinct and unanticipated fine structure, which is clear and replicable in each subject individually: the language-selective region appears as an island, with both more anterior and more posterior regions of domain-general activity.

Our data provide new insight into the complex functional structure of Broca’s area. The findings are consistent with and amplify previously reported structural heterogeneity of this brain region (e.g., [14–18]) and help resolve the longstanding debate about whether Broca’s area is language-specific or domain-general: our data show that it is both, in different subregions. In this light, the complexity of “Broca’s aphasia” is unsurprising, because lesions to this brain region will generally affect both linguistic and more domain-general functions.

Our findings open up myriad opportunities for future research. Does the functional subdivision of Broca’s area described here correspond to cytoarchitectonic/connectomic subdivisions of the same region, and how does it relate to nearby regions like BA9, BA46, and anterior insula [14–18]? What precise computations are conducted in the language-selective subregion? In the domain-general subregions? The latter two questions should replace the old question of the function of Broca’s area (e.g., [19]), because clearly Broca’s area is not a homogeneous functional unit. How does this functional subdivision account for the systematic pattern of preservation and loss in Broca’s aphasia? Which of the many previous functional responses that have been attributed to Broca’s area—from processing syntactic complexity to imitation—arise in the language subregion, and which in the domain-general subregions?

Beyond their implications for Broca’s area and for language processing, our findings offer a satisfying answer to classic questions about functional specificity in the frontal lobe, and cerebral cortex more generally: The human brain contains both regions that are highly specialized for a particular domain and regions that are broadly engaged by a wide range of stimuli and tasks. An important goal for future research will be to understand how brain regions with such starkly different functional profiles work together to produce uniquely human cognition.

## Experimental Procedures

### Participants

Forty right-handed native English-speaking adults (28 females, 12 males) from the Massachusetts Institute of Technology (MIT) community were paid for their participation. All participants gave informed consent in accordance with the Internal Review Board at MIT. (This data set partially overlaps with the data set reported in [13].)

### Design and Procedure

Each participant was scanned while performing the sentences/nonwords reading task and one or more of the other six tasks (arithmetic addition, spatial/verbal working memory, two versions of the multisource interference task [MSIT] and the classical Stroop task; see [13] for detailed descriptions of the tasks). Between 13 and 16 participants performed each of these six tasks.

### fMRI Data Acquisition

Structural and functional data were collected on a whole-body Siemens 3T Trio scanner with a 32-channel head coil at the Athinoula A. Martinos Imaging Center at the McGovern Institute for Brain Research at MIT. T1-weighted structural images were collected in 176 sagittal slices with 1 mm isotropic voxels (time of repetition [TR] = 2,530 ms, time of echo [TE] = 3.48 ms). Functional data were acquired using an echo planar imaging sequence with a 90% flip angle and using generalized autocalibrating partially parallel acquisition (GRAPPA) with an acceleration factor of 2 in 31 near-axial interleaved slices (in-plane resolution 2.1 × 2.1, slice thickness 4 mm; distance factor 10%; field of view 200 mm) with TR = 2,000 ms and TE = 30 ms. The first 10 s of each run was excluded to allow for steady-state magnetization. The scanning session included several functional runs, with each run containing a mixture of hard and easy blocks, counterbalanced for order.

### Statistical Analyses

MRI data were analyzed using SPM5 (http://www.fil.ion.ucl.ac.uk/spm) and custom MATLAB scripts (available from http://web.mit.edu/evelina9/www/funcloc.html and http://www.nitrc.org/projects/spm_ss). Each subject’s data were motion corrected and then normalized in a common brain space (Montreal Neurological Institute [MNI] template) and resampled into 2 mm isotropic voxels. Data were then smoothed using a 4 mm Gaussian filter and high-pass filtered (at 200 s).

### Defining Broca’s Area

Defining Broca’s area in vivo is a challenge because intersubject macroanatomical variability (including the sizes, shapes, and locations of gyri and the depths, detailed branching patterns, and locations of sulci; [20–26]) leads to poor alignment of anatomical features across brains in a common stereotaxic space. Furthermore, cytoarchitectonic zones, including BAs 44 and 45, have been shown to have poor alignment with macroanatomy in the frontal lobes (e.g., [14, 27–29]). Consequently, even at the level of individual subjects—using native anatomy—defining Broca’s area based on the anatomical landmarks is problematic. As a result, we used the most common way of defining Broca’s area in fMRI studies. Specifically, we used estimates of BA44 and BA45 in the MNI stereotaxic space with the help of the wfu_pickatlas tool [11]. Each of these region of interest (ROI) masks was then intersected with each individual subject’s activation map for (1) the sentences > nonwords contrast and (2) the nonwords > sentences contrast, each thresholded at p < 0.001 uncorrected level, to define each subject’s functional ROIs (fROIs) (no spatial contiguity constraints were imposed on these fROIs; any voxel that passed the specified threshold and fell within the boundaries of the anatomical parcel was included in the fROI definition).

To estimate the responses of these fROIs to various conditions, we averaged the responses across the voxels in each subject’s individually defined fROI and then averaged these values across subjects for each region. To estimate the responses to sentences and nonwords, we used all but the first run to define the fROIs and the first run to estimate the responses, so that all the data used to estimate response magnitudes were independent of the data used for ROI definition. One-tailed t tests were performed to evaluate the hypotheses: sentences > nonwords and nonwords > sentences for the language-selective and domain-general fROIs, respectively, and hard > easy for each of the other tasks in both kinds of fROIs.

## Figures and Tables

**Table 1:** Effect Sizes and Associated Statistics for the Effects Shown in Figure 2

	Effect Size (SE)	Degrees of Freedom	t Value	p Value
**Language-Selective fROI within BA45**				

Localizer	0.35 (0.08)	38	4.53	<0.0001
Math H > E	−0.02 (0.09)	12	−0.22	n.s.
Spatial WM H > E	−0.33 (0.11)	15	−3.10	n.s.
Verbal WM H > E	−0.04 (0.12)	12	−0.31	n.s.
MSIT H > E	−0.12 (0.07)	14	−1.72	n.s.
vMSIT H > E	−0.20 (0.08)	13	−2.30	n.s.
Stroop H > E	0.08 (0.07)	13	1.07	n.s.

**Language-Selective fROI within BA44**				

Localizer	0.32 (0.10)	36	3.34	<0.001
Math H > E	−0.004 (0.08)	11	−0.1	n.s.
Spatial WM H > E	−0.15 (0.09)	14	−1.61	n.s.
Verbal WM H > E	0.28 (0.10)	10	2.90	<0.01
MSIT H > E	−0.04 (0.07)	14	−0.60	n.s.
vMSIT H > E	−0.005 (0.11)	11	0.04	n.s.
Stroop H > E	0.28 (0.07)	12	4.02	<0.001

**Domain-General fROI within BA45**				

Localizer	0.38 (0.09)	36	4.32	<0.0001
Math H > E	0.49 (0.17)	11	2.87	<0.01
Spatial WM H > E	0.46 (0.17)	14	2.69	<0.01
Verbal WM H > E	0.11 (0.13)	10	0.87	n.s.
MSIT H > E	0.24 (0.11)	13	2.10	<0.05
vMSIT H > E	0.49 (0.10)	11	5.11	<0.001
Stroop H > E	0.40 (0.12)	12	3.51	<0.01

**Domain-General fROI within BA44**				

Localizer	0.35 (0.08)	35	4.30	<0.0001
Math H > E	0.22 (0.10)	12	2.17	<0.05
Spatial WM H > E	0.27 (0.13)	15	2.07	<0.05
Verbal WM H > E	0.32 (0.10)	9	3.23	<0.01
MSIT H > E	0.31 (0.10)	13	3.04	<0.01
vMSIT H > E	0.40 (0.10)	11	3.94	<0.01
Stroop H > E	0.33 (0.10)	11	3.31	<0.01

Effect sizes (in percent blood oxygen level-dependent signal change) are given with SE in parentheses, and the associated statistics for the effects shown in Figure 2. Note that the localizer contrast for the language-selective functional ROIs (fROIs) is sentences > nonwords, and for the domain-general fROIs it is nonwords > sentences. Note that hard > easy (H > E) tests were one-tailed, so that only positive differences are marked as significant. n.s., not significant.
